# Evaluation of Holmium(III), Erbium(III), and Gadolinium(III) Accumulation by Cyanobacteria *Arthrospira platensis* Using Neutron Activation Analysis and Elements’ Effects on Biomass Quantity and Biochemical Composition

**DOI:** 10.3390/microorganisms12010122

**Published:** 2024-01-07

**Authors:** Inga Zinicovscaia, Liliana Cepoi, Ludmila Rudi, Tatiana Chiriac, Dmitrii Grozdov

**Affiliations:** 1 Joint Institute for Nuclear Research, 6 Joliot-Curie Str., 141980 Dubna, Russia; dsgrozdov@rambler.ru; 2 Horia Hulubei National Institute for R&D in Physics and Nuclear Engineering, 30 Reactorului Str., 077125 Măgurele, Romania; 3 Institute of Microbiology and Biotechnology, Technical University of Moldova, 1 Academiei Str., 2028 Chisinau, Moldova; liliana.cepoi@imb.utm.md (L.C.); ludmila.rudi@imb.utm.md (L.R.); tatiana.chiriac@imb.utm.md (T.C.)

**Keywords:** rare-earth elements, *Arthrospira platensis*, holmium, erbium, gadolinium, neutron activation analysis

## Abstract

Rare-earth elements are released into the aquatic environment as a result of their extensive use in industry and agriculture, and they can be harmful for living organisms. The effects of holmium(III), erbium(III), and gadolinium(III) when added to a growth medium in concentrations ranging from 10 to 30 mg/L on the accumulation ability and biochemical composition of *Arthrospira platensis* were studied. According to the results of a neutron activation analysis, the uptake of elements by cyanobacteria occurred in a dose-dependent manner. The addition of gadolinium(III) to the growth medium did not significantly affect the amount of biomass, whereas erbium(III) and holmium(III) reduced it up to 22% compared to the control. The effects of rare-earth elements on the content of proteins, carbohydrates, phycobiliproteins, lipids, β carotene, and chlorophyll *a* were evaluated. The studied elements had different effects on the primary biomolecule content, suggesting that holmium(III) and erbium(III) were more toxic than Gd(III) for *Arthrospira platensis*.

## 1. Introduction

A collection of 17 elements known as rare-earth elements (REEs) includes 15 lanthanides (La, Ce, Pr, Nd, Pm, Sm, Eu, Gd, Tb, Dy, Ho, Er, Tm, Yu, and Lu), as well as Sc and Y [1,2]. The increased use of REEs in agriculture, metallurgy, the nuclear and chemical industries, and the production of magnets, luminescent and laser materials, superconductors, batteries, smartphones, and electronics is determined by their unique chemical, electrical, magnetic, and optical properties [3,4,5].

The widespread use of REEs in various industries causes their release in soil, water bodies, and the atmosphere at concentrations that are frequently orders of magnitude higher than the natural background levels [6,7], resulting in environmental contamination, which may have a serious negative impact on human health [6]. Thus, it was reported that endomyocardial fibrosis, lung and bladder cancer, indigestion, diarrhea, abdominal distension, anorexia, weakness, fatigue, etc. were all linked to high concentrations of REEs in humans [8,9]. 

Depending on the element, its quantity, and the organisms exposed, REEs can have either harmful or stimulating effects on aquatic organisms. Eight doped lanthanide-based ceramic oxides were tested on crustaceans and duckweeds, and either very low acute toxicity or no toxicity were seen [10]. Bergsten-Torralba et al. [11] demonstrated the toxicity of La, Nd, Sm, and their combinations for algae, microcrustaceans, and fungi. Several studies have reported the toxicity of Ce in nanoform for microalgae [12,13]. 

Among the 17 REEs, the influence of La, Ce, Gd, and Y on aquatic organisms has primarily been researched [12,14,15,16,17]. Despite studies and review papers detailing the bioaccumulation of REEs by aquatic organisms, their uptake by and effect on cyanobacteria have scarcely been investigated. According to numerous studies [7,18,19,20], cyanobacteria, including *Arthrospira platensis*, have a high biosorption and bioaccumulation capacity for heavy metals. This makes them promising candidates for polluted water bodies and wastewater remediation. 

In recent years, the cyanobacterium *Arthrospira platensis* has primarily been used as a biosorbent to recover REEs from solutions. Two strains of *Spirulina platensis* were applied to recover Nd from batch solutions. Both strains showed high adsorption capacity: 72.5 mg/g for the LEB-18 strain and 48.2 mg/g for LEB-52 [21]. Approximately 80% of Yb ions were adsorbed by spirulina from simulated mine wastewater [22], and 87.62% of the ions were absorbed from an aqueous solution [23]. Endemic and commercial strains of *Arthrospira platensis* were able to recover 18.1 and 38.2 mg/g, respectively, of Ce ions [24]. *Arthrospira platensis* showed a high adsorption capacity of 89.5 mg/g for Eu ions [25]. 

It is worth noting that little research has been done on their ability to bioaccumulate these elements. Thus, Zinicovscaia and co-authors [26] investigated the uptake of Dy, Sm, Tb, La, Nd, and Yb by spirulina biomass. The accumulation of Eu and Y ions by *Arthrospira platensis* and their effects on the biochemical composition of the biomass were reported in [25,27]. At the same time, the bioaccumulation technique, which allows the uptake of high levels of pollutants and their biotransformation into less harmful forms, is more appropriate for in situ remediation [28]. In this case, it is crucial to maintain the optimal level of biomass productivity and its biochemical composition in order to guarantee the excellent bioaccumulation potential of cyanobacteria. 

In the current study, neutron activation analysis was used to examine the accumulation of gadolinium(III) (one of the most studied REEs), holmium(III), and erbium(III) (two less well-examined elements) by the cyanobacterium *Arthrospira platensis*. The effects of REEs on the biomass, biochemical composition, and antioxidant activity of *Arthrospira platensis* were evaluated. As far as we are aware, this is the first study to report the effects of the aforementioned REEs on *Arthrospira platensis.*

## 2. Materials and Methods

### 2.1. Chemicals and Object of Study

The following salts were used to prepare experimental solutions with element concentrations of 10–30 mg/L: Er(NO_3_)_3_·6H_2_O, Gd(NO_3_)_3_·6H_2_O, and Ho(NO_3_)_3_·5H_2_O (analytical grade, Merck, Darmstadt, Germany). The metal concentrations in the solutions were determined using an ICP-OES spectrometer (PlasmaQuant PQ 9000 Elite, Analytik Jena, Jena, Germany). The study’s subject was the cyanobacterium *Arthrospira platensis* (*A. platensis*) CNMN -CB-02, which was obtained from the Institute of Microbiology and Biotechnology of the Technical University of Moldova in Chisinau, Republic of Moldova.

### 2.2. Experimental Design

*A. platensis* biomass was cultivated in a growth medium that contained the following components (g/L): NaNO_3_—2.5; NaHCO_3_—8.0; NaCl—1.0; K_2_SO_4_—1.0; MgSO_4_∙7H_2_O—0.2; CaCl_2_—0.024; FeSO_4_—0.01; EDTA—0.08; H_3_BO_3_—0.00286; MnCl_2_∙4H_2_O—0.00181; ZnSO_4_∙7H_2_O—0.00022; CuSO_4_∙5H_2_O—0.00008; MoO_3_—0.000015. Holmium(III), erbium (III), and gadolinium(III) were added in concentrations varying from 10 to 30 mg/L to the growth medium on the first day of biomass growth (the inoculum concentration was 0.4 g/L), and its cultivation continued for the next five days. The biomass was grown in Erlenmeyer flasks with a working volume of 500 mL under constant conditions—temperature: 30 °C, pH: 10.0, and light intensity: 55 µM photons/m^2^/s. The biomass was shaken two times per day at an interval of 12 h for 45 min with a frequency of 100 rpm on a UNIMAX 1010 shaker (Heidolph, Schwabach, Germany). 

The biomass was separated from the medium through filtration using a 5–8 µm “white ribbon filter” and was divided into two parts; one was dried and used for neutron activation analysis. while the other was frozen and utilized for biochemical analyses. As a control, biomass was grown without REEs. 

### 2.3. Neutron Activation Analysis (NAA)

Instrumental neutron activation analysis at the REGATA facility of the IBR-2 reactor (JINR, Dubna) was used to evaluate the uptake of Ho, Er, and Gd by the biomass. After irradiation for three days at a neutron flux of 1.1 × 10^11^ cm^−2^ s^−1^, the samples were repacked and measured after 20 days of decay for 2 h. The gamma spectra of the samples were measured using HPGe detectors with a relative efficiency of 100% and a resolution of 1.8–2.0 keV for a total absorption peak of 1332 keV of the isotope ^60^Co. The gamma spectra were stored, displayed, and analyzed using the Genie 2000 software, version 3.4, and the metal concentrations were computed using the “Concentrations” software, version 1.8. 

Using standard reference materials from the National Institute of Standards and Technology (NIST SRM 1547—peach leaves—and NIST SRM 1632c—trace elements in coal (bituminous coal)), the quality control of the measurements was ensured. The experimental and certified values were in good agreement. 

### 2.4. Biomass Quantity

The amount of biomass was calculated indirectly by measuring the absorbance of the *A. platensis* suspension at 750 nm using a T80 UV/VIS spectrophotometer (PG Instruments Ltd., Alma Park, Woodway Lan, Wibtoft, Leicestershire, UK), and the result was expressed in g/L. The quantity of biomass was computed based on the correlation between the absorbance of the biomass suspension and the mass of the cell fraction in the suspension. More details can be found in [29]. 

### 2.5. Biochemical Analysis and Determination of the Antioxidant Activity 

The content of protein was estimated by applying the Lowry method, which was based on the formation of a copper–protein complex in an alkaline environment. The spectrophotometric analysis described by Siegelman and Kycia was used to assess the amount of phytobiliproteins in a water extract. Based on the interaction of the carbohydrates with the Anthrone reagent (C_14_H_10_O) in acid media, the carbohydrates were identified using a spectrophotometric technique. A spectrophotometric determination of the lipid content was performed on the basis of lipid degradation using a phospho-vanillin reagent. The content of chlorophyll a and β-carotene was measured in an ethanolic extract. The absorbance of chlorophyll a was measured at 665 nm with an extinction coefficient of 0.8 × 10^5^ M^−1^cm^−1^, and that of β-carotene was measured at 450 nm with an extinction coefficient of 1.5 × 10^5^ M^−1^cm^−1^. The biochemical analysis is described in detail in [29]. The above-mentioned biomolecules’ content was reported as the percentage of the absolute dry biomass. The content of malondialdehyde (MDA) was ascertained spectrophotometrically by analyzing the reactive products of thiobarbituric acid [29]. 

### 2.6. Antioxidant Activity According to the ABTS^+^ Radical Cation Assay 

By using the ABTS+ (2.2 azinobis 3-ethylbenzothiazoline-6-sulfonic acid) radical cation decolorization assay, the antioxidant activity of the water extracts was determined.

### 2.7. Statistical Analysis 

The experiments were performed in triplicate, and the average values were used for the discussion of the results. Student’s *t*-tests were applied to reveal differences between the control and experimental samples.

## 3. Results and Discussion

### Element Uptake by A. platensis Biomass

**Figure 1:**
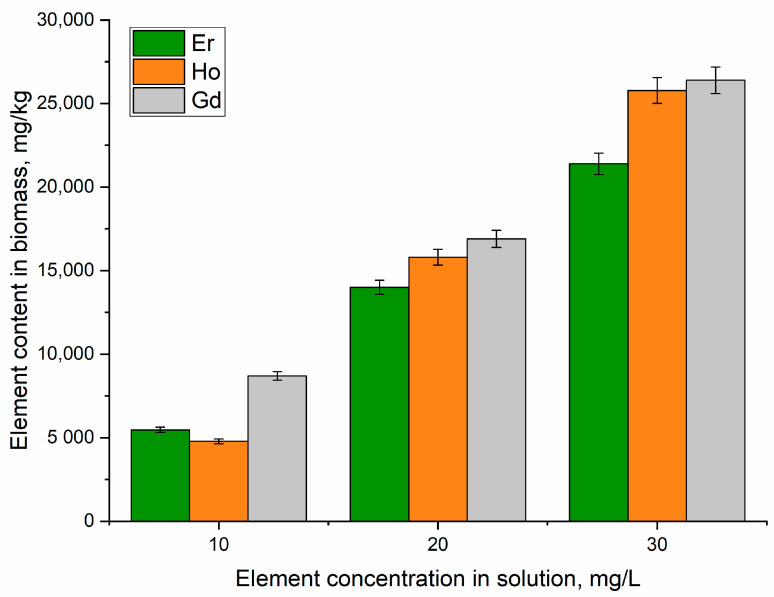
Uptake of Er, Ho, and Gd by *A. platensis* biomass cultivated in a medium supplemented with REEs.

The biological activity of REEs in microorganisms can be explained by the fact that their ionic radii are similar to those of essential elements, including Ca, Mn, Mg, Fe, and Zn. This could cause the polymerization of macromolecules or the replacement of vital metal ions from their ion-binding proteins. The particular permeability of the cellular membranes may change as a result of the chelation of REEs with the ion-binding sites of proteins and ion channels, leading to a shortage or excess of ions in the intracellular and extracellular voids [1]. According to the results of the NAA, *A. platensis* demonstrated a relatively high accumulation capacity for the studied metal ions, which were in the following order: gadolinium(III) > holmium(III) > erbium(III) (Figure 1). Thus, *A. platensis* accumulated 5470–21,400 mg/kg of Er, 4780–25,780 mg/kg of Ho, and 8600–27,400 mg/kg of Gd. 

When used to accumulate REEs, actinomycetes and Gram-positive bacteria showed higher preference for Eu than for Gd [30]. In a previously performed study [26], it was shown that *A. platensis* had a high affinity for lanthanum and dysprosium, a moderate affinity for neodymium and samarium, and a low affinity for terbium and ytterbium. It should be mentioned that REE uptake occurred in a dose-dependent manner; the higher the concentration of the element in the medium, the greater its accumulation in the biomass. 

**Figure 2:**
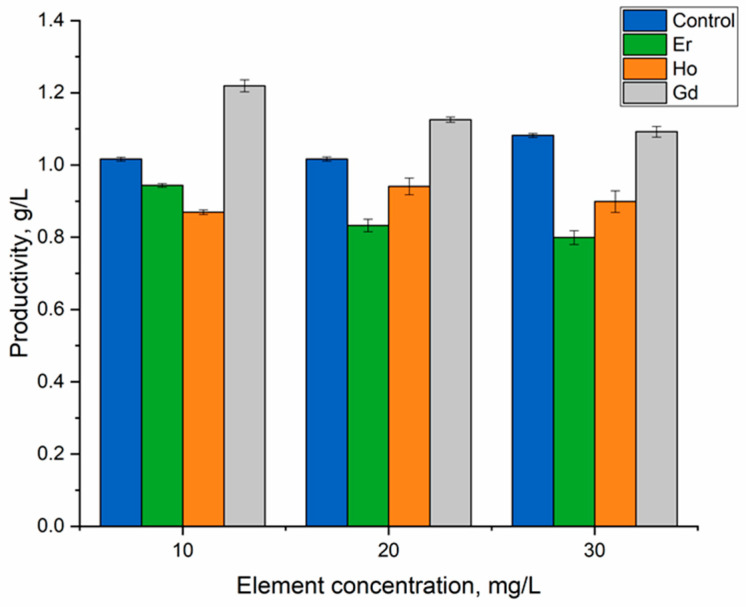
Effects of holmium(III), erbium(III), and gadolinium(III) on *A. platensis* productivity when introduced into the cultivation medium at different concentrations.

Different effects of the REEs on biomass productivity were seen (Figure 2). At concentrations of 10 mg/L and 20 mg/L, gadolinium(III) stimulated an increase in biomass productivity relative to the control by 20% and 10%, respectively. The biomass productivity was at the level of the control at the maximum applied concentration. Erbium(III) and holmium(III) inhibited biomass growth. All tested concentrations of erbium(III) led to a decrease in the amount of biomass, from 7.1% at a concentration of 10 mg/L (*p* = 0.00088) to 21.3% at a concentration of 30 mg/L (*p* = 0.002). In the case of holmium(III), the decrease in the amount of biomass was more modest and reached 13.7% at a metal concentration of 10 mg/L (*p* = 0.0002).

Lanthanum and dysprosium stimulated the growth of spirulina biomass productivity, neodymium and samarium maintained it at the level of the control biomass, and terbium and ytterbium reduced the biomass [26]. Europium influenced spirulina biomass only at high metal concentrations [25]. 

**Figure 3:**
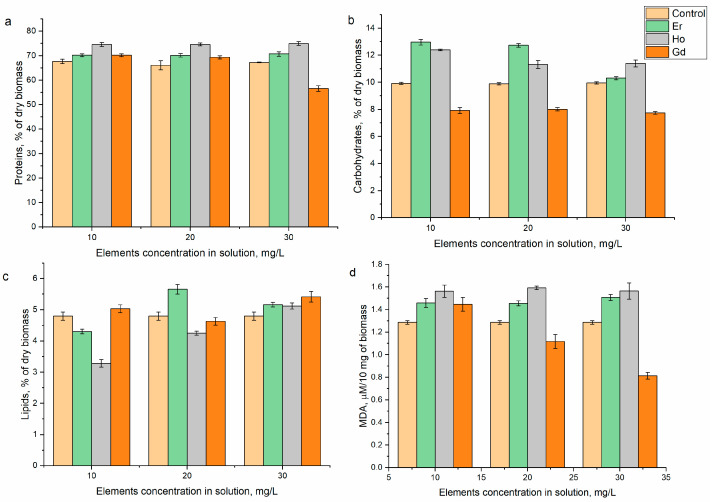
Effects of holmium(III), erbium(III), and gadolinium(III) on the content of (**a**) proteins, (**b**) carbohydrates, (**c**) lipids, and (**d**) MDA in *A. platensis* biomass when introduced into the cultivation medium at different concentrations.

The lanthanides influenced the content of proteins in different ways (Figure 3a). As a result, the addition of erbium(III) to the medium did not change the content of proteins; the latter remained on the level of the control. Holmium(III) caused a 10% increase in protein content compared to the control; this difference was statistically significant at concentrations of 10 and 20 mg/L (*p* = 0.0022 and 0.0029, respectively) and suggests an impairment of physiological processes in the biomass. The protein content was unaffected by the addition of gadolinium(III) to the medium at concentrations of 10 and 20 mg/L, but at 30 mg/L, it dropped by roughly 16% in comparison with the control. The presence of lanthanum, dysprosium, neodymium, samarium, terbium, and ytterbium did not significantly affect the content of proteins in the spirulina biomass, while europium and yttrium led to its decrease [25,26,27]. 

The supplementation of the cultivation medium with erbium(III) resulted in an increase in the content of carbohydrates in comparison with the control (Figure 3b) by 30.2% at an element concentration of 10 mg/L and by 27.9% at a concentration of 20 mg/L (*p* = 0.0015 and 0.0007, respectively). The stimulatory effect on the carbohydrates was reduced at a concentration of 30 mg/L, with their content remaining at the control levels. The amount of carbohydrates in the *A. platensis* biomass exceeded the amount in the control by 12.1 to 24.5% when holmium(III) was present in the growth medium, which can be explained by the activation of the protective mechanisms and elimination of toxic elements from the cells. The same pattern was observed when spirulina was grown in the presence of samarium, lanthanum, dysprosium, and terbium [26]. Gadolinium(III) had the opposite effect, causing a decrease in the carbohydrate content by 19–22% in comparison to the control. Neodymium, ytterbium, and europium reduced the content of carbohydrates in spirulina biomass [25,26,27].

There was no discernible pattern in the REEs’ impacts on the lipid content (Figure 3c). When erbium(III) was present in the medium at a concentration of 10 mg/L, the amount of lipids decreased by 10.3% (*p* = 0.007) compared to the control, while at a concentration of 20 mg/L. their content was already 17% higher (*p* = 0.02). The difference between the control and experimental samples was less than 8% at an erbium(III) concentration of 30 mg/L. The negative effect of holmium(III) on the content of lipids decreased with the increase in its concentration in the medium. As a result, the content of lipids decreased by 31.5% (*p* = 0.007) compared to the control at a holmium(III) concentration of 10 mg/L and by only 6.7% at a concentration of 30 mg/L. The content of lipids in the control and experimental samples was similar at gadolinium(III) concentrations of 10 and 20 mg/L, but at 30 mg/L, their content in the experimental samples increased by 12%. The effects of other REEs on the lipid content in spirulina biomass were also very different [25,26,27].

The quantity of lipid oxidative degradation products (MDA) can be used to determine the presence of oxidative stress. The presence of lanthanides in the cultivation medium caused a change in the level of MDA in the biomass (Figure 3d). In the case of gadolinium(III), at all concentrations, the amount of MDA was lower than or roughly equivalent to that in the control biomass. At all applied element concentrations, a measurable increase in the MDA level was seen for erbium(III) and holmium(III). Thus, the addition of erbium(III) to the medium resulted in an increase in MDA by 13.4–17.1% compared to the control (*p* < 0.01) and in holmium (III) by 21.6–23.7% (*p* < 0.05), which showed the toxic effects of metal ions on spirulina biomass.

**Figure 4:**
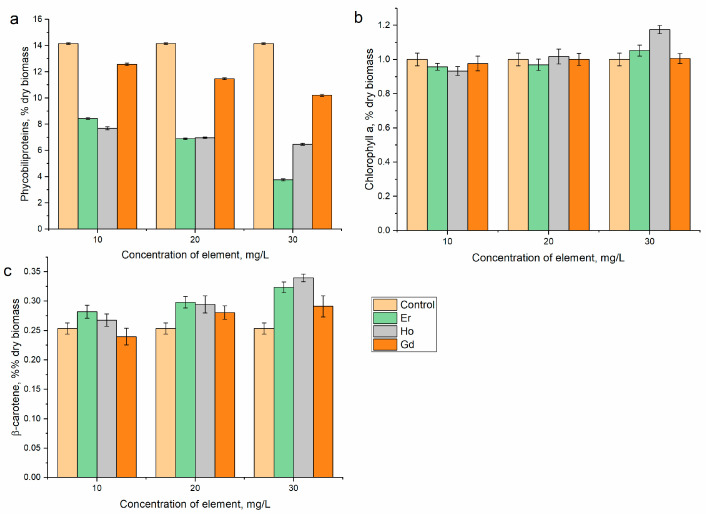
Effects of holmium(III), erbium(III), and gadolinium(III) on the content of (**a**) phycobiliproteins, (**b**) chlorophyll a, and (**c**) β-carotene in *A. platensis* biomass when introduced into the cultivation medium at different concentrations.

The content of phycobiliproteins in *A. platensis* biomass was significantly modified under the influence of the lanthanides (Figure 4a). All three elements reduced the content of the pigment. and with the increase in the elements’ concentrations, the adverse effect was intensified. It was observed that the content of phycobiliproteins was reduced twice or more in the experimental variants in which the content of carbohydrates was at the level of the control or exceeded it. Thus, in the case of erbium(III), their content decreased by 40.1–73.3%, and under the action of holmium(III), it decreased by 45.3–54.1%. The level of phycobiliprotein reduction was lower—10.8–27.6%—in the medium supplemented with gadolinium(III), where the content of carbohydrates in the spirulina biomass significantly decreased. A decrease in the level of phycobiliproteins in spirulina biomass was observed in the case of other REEs as well, and it could be associated with a reduction in the efficiency of photosynthesis with a decrease in the antioxidant capacity of the biomass [25,26,27].

The content of chlorophyll a in *A. platensis* biomass was a fairly stable parameter. Under the influence of the lanthanides, only some quantitative fluctuations were recorded (Figure 4b). Thus, holmium(III) at a concentration of 30 mg/L caused an increase in this content in the biomass by 17.4% (*p* < 0.05). In all other experimental variants, the content of chlorophyll did not differ significantly from the control. The total amount of chlorophyll in *Desmodesmus quadricauda* increased under the influence of gadolinium ions [31]. The content of β-carotene in *A. platensis* was modified under the influence of the lanthanides. As a result of the effects of erbium(III) and holmium(III), it increased at all applied concentrations by 11–28% and 5.6–34%, respectively (Figure 4c). In the case of gadolinium(III) at a concentration of 10 mg/L, it was reduced by 5.4% compared to the control, but at concentrations of 20 and 30 mg/L, it exceeded the control value by 10.7 and 15%, respectively. A similar pattern was observed for spirulina grown in a medium supplemented with other REEs and indicated the adaptation of the biomass to the presence of these elements in the medium [25,26,27]. The amounts of carotenoids and total lipids in the biomass of *Dunaliella salina* significantly increased when gadolinium nanoparticles were present in the growth medium [32]. A considerable increase in chlorophyll and carotenoid content in the miroalgae *Trachydiscus minutus* and *Parachlorella kessleri* compared to the control was observed in the presence of Gd(III) [33]. 

**Figure 5:**
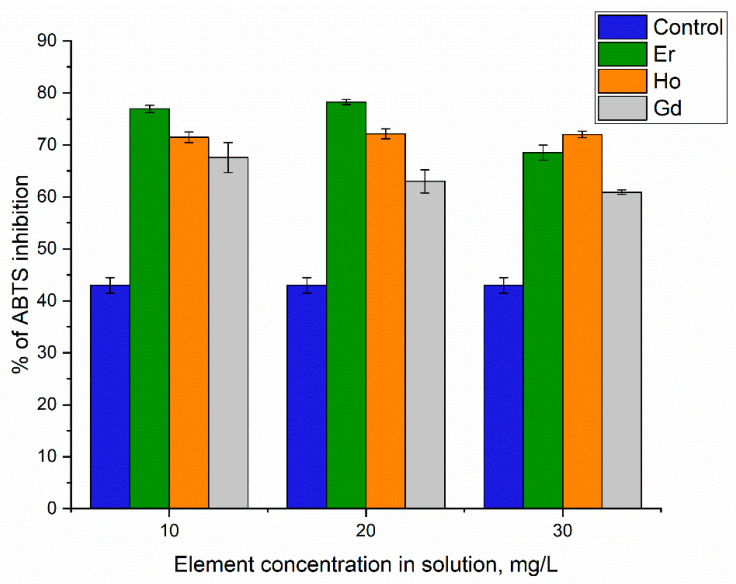
Effects of holmium(III), erbium(III), and gadolinium(III) on antiradical activity in water extracts obtained from *A. platensis* when introduced into the cultivation medium at different concentrations.

Numerous studies have documented the alteration of antioxidant activity in biomasses of microalgae and cyanobacteria under stress conditions. Thus, *Phaeodactylum tricornutum*, *Tetraselmis suecica*, and *Chlorella vulgaris* biomasses’ antioxidant activity could be altered through nutrient limitation according to [1]. The addition of lanthanides to the culture medium caused moderate stress conditions, which were indicated by an increase in the antioxidant activity of the water extract obtained from the *A. platensis* biomass (Figure 5).

Samarium, terbium, lanthanum, and dysprosium resulted in an increase in the antiradical activity of the spirulina biomass, indicating its tolerance to REEs. A decrease in the antioxidant activity of a biomass in the presence of ytterbium and neodymium showed its low tolerance to the mentioned elements [26].

## 4. Conclusions

The effects of different concentrations of erbium(III), gadolinium(III), and holmium(III) on the uptake capacity and biochemical composition of the cyanobacteria *Arthrospira platensis* were studied. Element accumulation was assessed using a neutron activation analysis. The highest accumulation capacity was obtained for Gd, followed by Ho and Er. The accumulation of gadolinium(III) did not provoke a significant impact on the biomass productivity or content of proteins, chlorophyll a, and β-carotene. The maintenance of the mentioned parameters on the level of the control biomass indicated the satisfactory physiological state of the culture under the conditions of contact with different concentrations of gadolinium(III). At the same time, important quantitative changes occurred in the content of carbohydrates and phycobiliproteins. The changes in these two parameters in *Arthrospira platensis* were associated with stress, or at least with a significant external impact. In the cases of erbium (III) and holmium(III), a decrease in the biomass productivity and the content of phycobiliproteins and an increase in the content of carbohydrates and MDA indicated the potential toxic effects of lanthanides. *Arthrospira platensis* can be applied for the remediation of water containing REEs in concentrations that do not cause toxic effects on biomass.

## Data Availability

Data are contained within the article.
